# A Bittersweet Response to Infection in Diabetes; Targeting Neutrophils to Modify Inflammation and Improve Host Immunity

**DOI:** 10.3389/fimmu.2021.678771

**Published:** 2021-06-03

**Authors:** Rebecca Dowey, Ahmed Iqbal, Simon R. Heller, Ian Sabroe, Lynne R. Prince

**Affiliations:** ^1^ Department of Infection, Immunity and Cardiovascular Disease, University of Sheffield, Sheffield, United Kingdom; ^2^ Sheffield Teaching Hospitals National Health Service (NHS) Foundation Trust, Sheffield, United Kingdom; ^3^ Department of Oncology and Metabolism, University of Sheffield, Sheffield, United Kingdom

**Keywords:** type 1 diabetes, type 2 diabetes, neutrophil, inflammation, infection, NETosis, hyperglycaemia, COVID-19

## Abstract

Chronic and recurrent infections occur commonly in both type 1 and type 2 diabetes (T1D, T2D) and increase patient morbidity and mortality. Neutrophils are professional phagocytes of the innate immune system that are critical in pathogen handling. Neutrophil responses to infection are dysregulated in diabetes, predominantly mediated by persistent hyperglycaemia; the chief biochemical abnormality in T1D and T2D. Therapeutically enhancing host immunity in diabetes to improve infection resolution is an expanding area of research. Individuals with diabetes are also at an increased risk of severe coronavirus disease 2019 (COVID-19), highlighting the need for re-invigorated and urgent focus on this field. The aim of this review is to explore the breadth of previous literature investigating neutrophil function in both T1D and T2D, in order to understand the complex neutrophil phenotype present in this disease and also to focus on the development of new therapies to improve aberrant neutrophil function in diabetes. Existing literature illustrates a dual neutrophil dysfunction in diabetes. Key pathogen handling mechanisms of neutrophil recruitment, chemotaxis, phagocytosis and intracellular reactive oxygen species (ROS) production are decreased in diabetes, weakening the immune response to infection. However, pro-inflammatory neutrophil pathways, mainly neutrophil extracellular trap (NET) formation, extracellular ROS generation and pro-inflammatory cytokine generation, are significantly upregulated, causing damage to the host and perpetuating inflammation. Reducing these proinflammatory outputs therapeutically is emerging as a credible strategy to improve infection resolution in diabetes, and also more recently COVID-19. Future research needs to drive forward the exploration of novel treatments to improve infection resolution in T1D and T2D to improve patient morbidity and mortality.

## Introduction

The number of people with diabetes (PWD) globally will exceed 500 million by 2035. Type 1 diabetes (T1D) is an autoimmune condition characterised by T-cell mediated pancreatic β cell destruction and absolute insulin deficiency (1). T1D represents up to 10% of all diabetes worldwide and a small percentage (<10% type 1B) of affected individuals have no evidence of autoimmunity with the pathogenesis being idiopathic (2, 3). A complex interplay of genetic, epigenetic, environmental, and immunologic factors is thought to contribute to the pathogenesis of T1D. Genome-wide association studies have identified more than 50 genetic risk loci to date but the main genes predisposing to T1D are located within the human leukocyte antigen (HLA) on chromosome 6 (4, 5). Alleles at the HLA locus account for up to 50% of cases with familial clustering (6–8). Epidemiological studies have implicated a number of environmental factors in the pathogenesis of T1D, including viruses and nutrients such as cow’s milk protein (4, 5). These factors are thought to trigger an autoimmune response consequent upon molecular mimicry in that pancreatic autoantigens that resemble viral or dietary epitopes undergo cellular destruction (9, 10). Pancreatic β cell destruction involves both cellular and humoral immunity. Autoreactive T-cells are thought to induce apoptosis in a pancreatic islet milieu rich in pro-inflammatory cytokines including IL-1, TNF-α, and IFN-*γ* (11). The presence of circulating autoantibodies against proinsulin and other autoantigens in β cells highlights the role of humoral immunity in disease pathogenesis. Indeed, circulating autoantibodies in T1D can occur before the biochemical and clinical manifestations and the presence of two or more autoantibodies in first-degree relatives strongly predicts clinical progression to T1D (12).

In type 2 diabetes (T2D), which accounts for 90-95% of all diabetes, a combined resistance to insulin both in skeletal muscle and the liver, in addition to defective insulin production by pancreatic β cells is present (13). In contrast to T1D, no predominant genetic locus has been found to increase susceptibility to T2D. Genomic studies reveal over 40 genetic variants that increase the risk of T2D, however, overall these genes account for 10% heritability (14, 15). A positive family history is important nonetheless with a 38% life-time risk of T2D in individuals who have one parent with T2D with this risk increasing to 60% if both parents have T2D (16, 17). In addition to multiple genes, environmental factors play a critical role in the pathogenesis of T2D. A sedentary lifestyle in addition to consumption of high-fat, high-calorie diets means the majority of individuals with T2D are overweight (6). Obesity related insulin resistance together with hypertension, dyslipidaemia, glucose intolerance, and eventually frank hyperglycaemia defines the metabolic syndrome and this clinical phenotype is commonly encountered in many people with T2D (18). Several mechanisms have been proposed to explain both insulin resistance in T2D which occurs early in the disease and pancreatic β cell dysfunction which is typically a late phenomenon. Increased levels of non-esterified fatty acids, pro-inflammatory cytokines, adipokines, and mitochondrial dysfunction are thought to drive insulin resistance (19). Progressive β cell failure is thought to occur due to glucotoxicity, lipotoxicity and direct cytotoxic effects from deposition of islet amyloid polypeptide (19). There is accumulating evidence that many of these mechanisms work in concert and are underpinned by low-grade activation of the innate immune system (20). This not only plays a part in the pathogenesis of T2D but is also causally linked to associated complications including dyslipidaemia and atherosclerosis (20). Elevated levels of pro-inflammatory cytokines including IL-6 and TNF-α and acute phase markers such as C-reactive protein are thought to disrupt insulin signalling although effects on glucose metabolism remain less clear (21). Humoral immunity may also play a part in the pathogenesis of T2D. Elevated serum gamma globulin levels, a nonspecific marker of humoral immune activation, have been associated with an increased risk of T2D in certain populations although the wider significance remains to be elucidated (22). Despite differences in pathophysiology, chronic hyperglycaemia is a fundamental biochemical abnormality present in both T1D and T2D, which is a key driver of aberrant neutrophil function. Increased susceptibility to infection is found in both types of diabetes, therefore this review will explore neutrophil function in the context of both T1D and T2D simultaneously.

PWD are at an increased risk of infection at various sites including skin and soft tissue (SSIs), urinary tract and the respiratory system (23, 24). Abscesses can be the first clinical presentation of diabetes in undiagnosed individuals, which occur before later vascular and neuropathic symptoms (25, 26). Infection resolution is often delayed and can lead to limb amputation in the lower extremities (27, 28). Reliance on antibiotic therapy means PWD receive increased prescriptions of antibiotics (29, 30). Antibiotic resistance is a global health concern and methicillin resistant *Staphylococcus aureus* (MRSA) was isolated in 15-30% of cases of diabetic foot disease (DFD), highlighting the importance of conservative antibiotic usage in this cohort and a need for new therapeutic strategies (31–33).

The innate immune system is dysregulated in both T1D and T2D (34, 35). Neutrophils are professional phagocytes of the host immune system and are critical in the clearance of pathogens, in particular *S. aureus*, which is the most common pathogen isolated in SSIs in PWD (36–38). Neutrophils are equipped with an arsenal of microbicidal effector functions. Upon activation in the circulation neutrophils migrate to sites of infection and inflammation by chemotaxis and respond to infection by engulfing pathogens *via* phagocytosis for intracellular killing by the release of cytotoxic granules and reactive oxygen species (ROS) (39). Neutrophils also release ROS, neutrophil extracellular traps (NETs) and granule proteins extracellularly in response to pathogens, all of which can damage host tissues (39, 40). Neutrophils are also central in co-ordinating the immune response to infection and produce a range of pro-inflammatory and anti-inflammatory cytokines which have autocrine and paracrine actions (39). Neutrophils rely predominantly on glucose as the sole energy source for the cell, and the impacts of hyperglycaemia and associated advanced glycation end products (AGEs) are the key causes of altered neutrophil function in T1D and T2D (41–45). T2D and obesity are also inherently linked, with increased circulating saturated fatty acids and the associated pro-inflammatory milieu also having immune-modulatory roles (46, 47).

Previous research investigating neutrophil function in T1D and T2D covers an expansive body of literature spanning 60 years, with every function of the neutrophil shown to be dysregulated in T1D or T2D. Early research in the field focused on neutrophil chemotaxis and phagocytosis, with the weight of evidence demonstrating a reduction of these functions in those with diabetes (48–51). There were some conflicting findings between early studies, perhaps caused by variations in participant selection and early experimental designs (52, 53). More recent research has predominantly focused on neutrophil ROS generation, pro-inflammatory cytokine production and aberrant neutrophil cell death mechanisms, which are proving to be critical mediators in the weakened response to infection in diabetes (54–57). Extracellular ROS production, pro-inflammatory cytokine release and NET formation are increased in diabetes, whereas neutrophil migration, apoptosis and intracellular ROS production are reduced, which ultimately impairs bacterial killing and inflammation (56, 58–61). Phenotypic variations in neutrophil function are supported by transcriptomic data, showcasing a fundamentally altered profile in key pro-inflammatory genes in neutrophils in PWD (62, 63).

Research aiming to therapeutically modify neutrophil function in response to infection in T1D or T2D lags compared to the volume of studies reporting observational differences between those with and without diabetes. However, research aiming to restore aberrant neutrophil function in diabetes is gaining momentum in the field, with a focus on modifying neutrophil ROS production and NETosis to improve infection outcomes (64–67). The enhanced susceptibility in PWD to COVID-19 infection has garnered global interest during the ongoing COVID-19 pandemic and approaches to improve neutrophil responses in people with diabetes might have important therapeutic potential (68–70). Furthermore, powerful stress responses during COVID-19 lead to hyperglycaemia and diabetic ketoacidosis among people with T2D, perhaps explaining in part their increased susceptibility to severe disease. Here, we will first explore the key drivers of neutrophil function in the diabetic microenvironment and then review key aspects of neutrophil function and how these critical functions are modified in diabetes. We then explore how these pathways have been therapeutically targeted to enhance infection clearance in diabetes, and highlight future important directions for research.

## Mediators of Neutrophil Function in the Diabetes Microenvironment

Hyperglycaemia is a key mediator of neutrophil dysfunction in T1D and T2D. Elevated blood glucose concentrations resulting from insulin insufficiency and tolerance is a core pathology of the disease. The impacts of hyperglycaemia on neutrophils are multi-factorial and present a complex interplay of dysregulated cellular mechanisms. Neutrophil metabolism is altered in response to excess glucose, to ensure intracellular glucose levels do not become toxic (42). Molecular shunting of glucose from glycolysis into the polyol and hexosamine pathway occurs (42, 71, 72). Metabolism *via* these pathways decreases levels of the intracellular ROS scavenger, glutathione and modifies transcription factors regulating pro-inflammatory genes (NF-κB, TGF-α, TGF-β) (42, 43, 71). Enhanced generation of cytokines further activates subsequent neutrophils, causing a feed forward loop of excessive inflammation in diabetes (73). Furthermore, hyperglycaemia causes *de novo* synthesis of the protein kinase C (PKC) activator, diacylglycerol (DAG), upregulating the formation of NADPH oxidase complex at the plasma membrane and leading to oxidative stress and NET formation (44, 71). Hyperglycaemia alters the osmolarity of the body fluids and hyperosmotic stress causes cell shrinkage and calcium influx into neutrophils, leading to derangements in phagocytosis and upregulation of pro-inflammatory cytokines (74, 75). High intracellular calcium concentrations deplete available ATP, impacting key energy dependant functions such as phagocytosis (74, 76). High glucose also impacts maturing neutrophils in the bone marrow. Hyperglycaemia induced myelopoiesis and leucocytosis in streptozotocin (STZ) and Akita mice (murine models of T1D) is mediated by the production of neutrophil alarmins s100 calcium proteins 8 and 9 (S1008/9) (77).

Hyperglycaemia upregulates the receptor for advanced glycation end products (RAGE) on the neutrophil cell surface (78). Advanced glycation end products (AGE) are formed from the non-enzymatic glycation of proteins (79). The pro-inflammatory impacts of AGE, which are extensively reviewed elsewhere, are of particular importance in mediating cardiovascular sequalae in diabetes (79–81). In brief, AGEs induce oxidative stress and pro-inflammatory gene expression (NF-κB) in multiple cell types, including neutrophils (42, 82, 83). AGE signals *via* the RAGE receptor on the neutrophil cell surface, which importantly is a multi-ligand receptor also for the alarmins S1008/9 and high-mobility group box 1 (HMGB1), further perpetuating inflammation (84, 85). Epigenetic modifications, which are the enzymatic alterations of chromatin to manipulate gene expression, were found in healthy murine macrophages co-incubated with AGE (86, 87). Increased methylation of NF-κB and enhanced cytokine transcription was subsequently found (86). Neutrophils display ‘metabolic memory’ in PWD, whereby modified cell phenotypes are maintained after the restoration of normoglycaemia, further prolonging deleterious effects (88–90). Investigation of epigenetic alterations of neutrophils in T1D and T2D is warranted to provide additional mechanistic understanding of the persisting neutrophil phenotype. Furthermore, whether hypoglycaemia or the oscillations between high and low blood glucose concentrations promotes neutrophil dysfunction is not yet known.

Glucose is not the only pro-inflammatory mediator increased in T1D and T2D. Lipid metabolism is altered in response to insulin deficiency and resistance, which increases lipogenesis and adipose tissue metabolism (91, 92). Circulating levels of free fatty acids and lipoproteins are increased in T1D and T2D, which can be further exacerbated by obesity and poor diet (93). The pro-inflammatory impacts of lipids and neutrophils are reviewed elsewhere and have shown to upregulate key pro-inflammatory neutrophil functions including cytokine generation and ROS production (91, 92, 94, 95). The negative impacts of ageing on neutrophil function is well documented, and also contributes to the neutrophil phenotype in older individuals with diabetes (96, 97).

**Figure 1:**
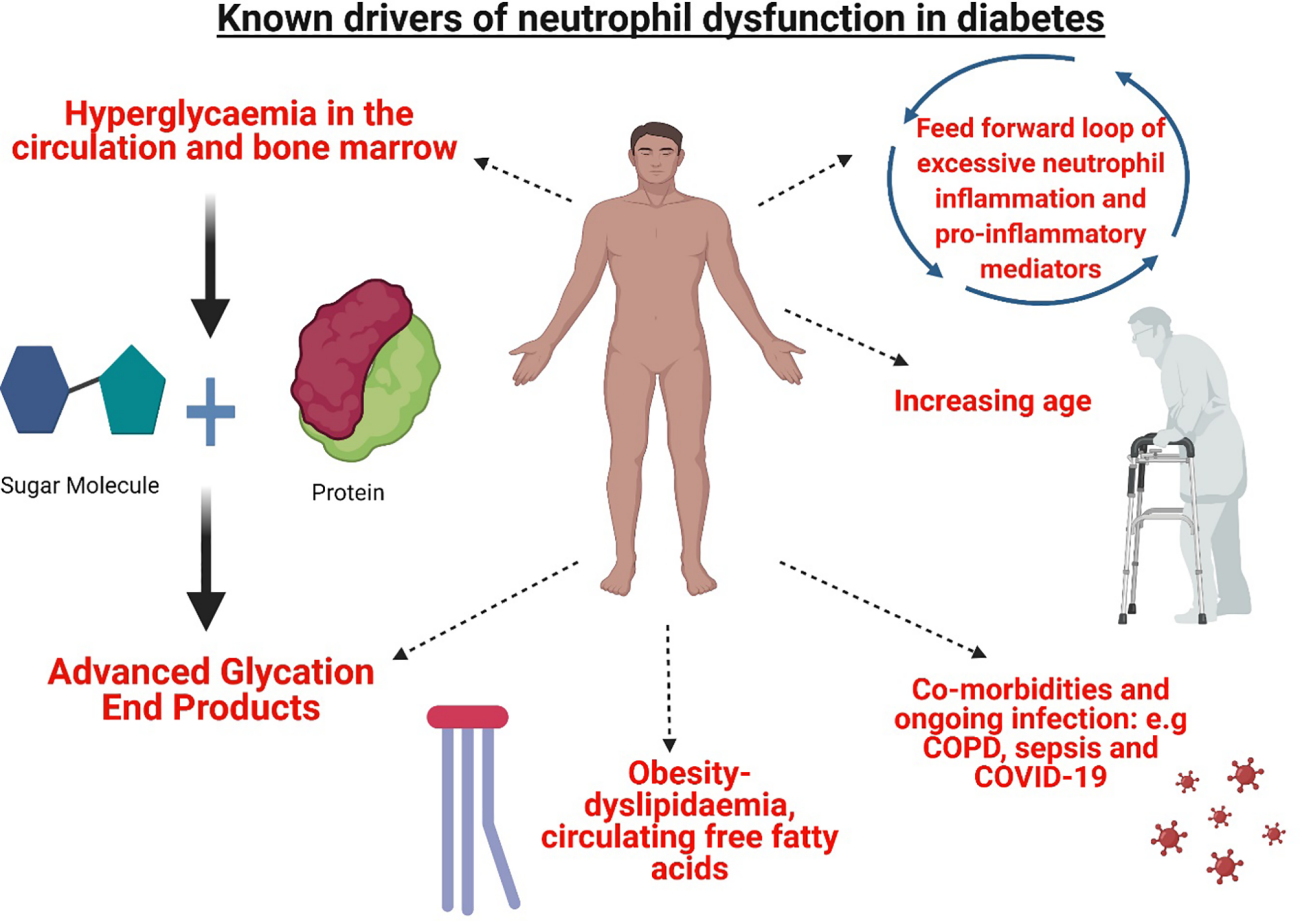
Mediators of neutrophil dysfunction present in T1D and T2D. The microenvironment of T1D and T2D presents a complex interplay of mediators of neutrophil dysfunction. Hyperglycaemia and the formation of advanced glycation end products in the circulation and the bone marrow modify circulating neutrophils and myeloid precursors. Metabolic perturbations in lipid metabolism and increased synthesis of circulating free fatty acids further contribute to aberrant dysfunction. Resulting activated neutrophils produce pro-inflammatory mediators adding to a cycle of inflammation. Increased age further impacts neutrophil function, in addition to co-morbidities and infection, where altered neutrophil functions are previously shown e.g chronic obstructive pulmonary disease (COPD) sepsis and COVID-19. Figure created with BioRender.com.

Despite what is already known about the influence of the diabetic microenvironment on neutrophil function, there are still potential drivers yet to be explored. Complement protein, C5a, is a potent anaphylatoxin and critical mediator of inflammation (98). Recent research demonstrates C5a was increased in the plasma of PWD and in murine models of both diabetes types (99). The impact of C5a on neutrophil function has not been directly investigated in the context of diabetes previously. However, there is a strong rationale to further investigate the role of C5a as neutrophil phagocytosis, phagosomal maturation, ROS production and apoptosis were impacted by C5a mediated signalling in patients with critical illness and sepsis and similar mechanisms may be of significant importance in diabetes (98, 100–102). Key mediators of neutrophil function in diabetes are summarised in **Figure 1**.

## Neutrophil Recruitment and Chemotaxis

Neutrophil transmigration from the circulation to the tissues in response to infection and inflammation is a well described process (103). In brief, circulating neutrophils respond to tissue derived signals for infection and injury which includes chemokines, damage associated molecular patterns (DAMPs) and bacterial products. This in turn triggers their interaction with the blood vessel wall *via* surface ligands to endothelial cell P and L selectins, facilitating the tethering and rolling of the neutrophil across the surface of the endothelium (104, 105). Sequential activation of neutrophils stimulates expression of integrins, slowing neutrophil rolling and facilitating neutrophil crawling along the endothelial cell surface, guided by chemoattractant gradients to the source of infection (104, 105). Neutrophils transmigrate predominantly through endothelial cell junctions into the tissue interstitial space and onwards to the site of injury or infection (103).

Chronically inflamed tissue such as in peripheral arterial disease, a common sequela of T2D, notably modifies the dynamics of neutrophil migration (106). Neutrophil recruitment to infection sites was reduced in multiple animal studies exploring infections caused by a range of pathogens, thus demonstrating migration to be a fundamental defect in neutrophil function in diabetes. They found neutrophil infiltration to the peritoneal cavity was reduced in alloxan treated mice (T1D model) with polymicrobial sepsis, in addition to reduced migration to the bladder in STZ treated mice with a UTI, caused by uropathogenic *Escherichia coli* (107, 108). Furthermore, reduced infiltration of neutrophils was demonstrated in a *S. aureus* hind paw infection in leptin deficient mice (murine T2D model) (109). Reduced neutrophil migration was associated with poor infection resolution and increased mortality across a number of studies (107–109).

There are multiple causative mechanisms for aberrant neutrophil migration with both the neutrophil and the endothelium shown to be altered in the diabetic microenvironment. Internalisation of the chemokine receptor CXCR2 was associated with reduced migration in a study of sepsis in mice with alloxan induced diabetes, which is a shared neutrophil dysfunction mechanism common to non-diabetes sepsis models (107, 110). CXCR2 expression is downregulated by TLR2 signalling, which involves G protein coupled receptor kinase-2 (GRK2) (111). TLR2 is activated by high glucose concentrations, AGEs, lipoproteins and DAMPS, which are released at increased levels in diabetes (55, 112, 113). Furthermore, the serum derived acute phase protein, α1-acid glycoprotein, upregulates GRK2 and further contributes to perturbations in neutrophil migration *in vivo* (107). Increased α1-acid glycoprotein concentration and glycosylation were found in people with T2D or with sepsis (114, 115). Administration of insulin reduced concentrations of α1-acid glycoprotein and restored neutrophil migration in a rodent model of sepsis and alloxan induced diabetes *in vivo* (107). Interestingly, Perieria et al. found an unknown serum protein from alloxan treated rats which inhibited chemotaxis *in vitro* and the activity of such was abolished by insulin, which one could speculate was α1-acid glycoprotein, although further studies are needed to corroborate this (116).

Neutrophil chemotaxis has been widely investigated *in vitro* in numerous studies using animal models and volunteers with T1D or T2D, with most of the research conducted in the 1970s - 1990s (**Table 1**). Despite some contradictory findings, which may be accountable by variations in study and experimental design, the burden of evidence suggests that neutrophil chemotaxis in diabetes is reduced (49, 51, 122). Early research using human volunteers found no correlation between increased blood glucose concentration and aberrant neutrophil chemotaxis, suggesting a reliance on existing blood glucose lowering agents may not be sufficient to restore chemotaxis in all individuals (48, 51, 118).

**Table 1:** Studies investigating neutrophil chemotaxis in diabetes.

Study	Animal model/human volunteer type	Chemotaxis phenotypes reported in diabetes
***Studies reporting a decrease in neutrophil chemotaxis in T1D or T2D compared to control***		
(48)	HVs + T2D volunteers	↓ in chemotaxis towards casein and human serum
(117)	HVs + 17 children with T1D	↓ chemotaxis towards *Staphylococcus epidermidis* & albumin
(118)	HVs + those with T2D (mild to severe periodontitis)	No difference between HVs and those with mild periodontitis+T2D. Significant ↓ in severe periodontitis + T2D. Endotoxin activated plasma and fMlp used as chemoattractant
(116)	Alloxan treated rat model	↓ in chemotaxis. Incubating healthy rat neutrophils in diabetic rat plasma also ↓ chemotaxis
(119)	HVs+ volunteers with T1D	↓ chemotaxis towards zymosan-activated plasma. No difference towards fMlp and *Escherichia coli* supernatant
(49)	HVs + people with T1D and T2D	↓ chemotaxis towards fMlp but no difference towards healthy control serum
(55)	Akita mouse (point mutation in Ins2 gene- inability to produce insulin-T1D model)	↓ chemotaxis towards fMlp and WKYMVm but no difference in random (unstimulated) migration.
(120)	Alloxan treated rats	No WT rats used in the study. ↓ chemotaxis towards casein and fMLP in rats with severe compared to mild diabetes
(121)	Neutrophils investigated from WT rats incubated in serum from Alloxan treated rats or WT	No difference in chemotaxis towards fMLP or Leukotriene B4. ↓ chemotaxis towards LPS-activated rat sera in diabetic serum group
(122)	HVs + people with T2D undergoing tooth extractions	↓ chemotaxis towards fMLP
(51)	HVs + people with insulin dependent diabetes	↓ chemotaxis towards fMLP
(123)	Low dose STZ-treated mice	↓ chemotaxis towards casein
***Studies reporting no difference in neutrophil chemotaxis in T1D or T2D compared to control***		
(124)	HVs + people with T1D or T2D). Mixture of children and adults in both groups.	No difference in chemotaxis to zymosan activated serum
(53)	HVs + those with T2D	No difference in chemotaxis towards fMLP
(125)	HVs + people with diabetes and periodontitis	No difference towards zymosan activated serum
(126)	HVs + people with T2D and periodontitis	No difference towards zymosan activated serum
(127)	HVs + people with T1D or T2D	No difference in chemotaxis towards fMLP

HVs, Healthy volunteers; fMlp, N-Formyl-methionyl-leucyl-phenylalanine; WT, Wild-type.

Despite a reduction in neutrophil migration and chemotaxis to infection, neutrophil activation measured by CD11b cell surface marker expression, and adhesion to the endothelium, were increased in both rodent models of diabetes and people with T2D *in vitro* (128–130). High glucose mediates increased neutrophil adhesion, by increasing expression of endothelial adhesion molecules (intracellular adhesion molecule-1, P-selectin and E-selectin), which was dependant on PKC signalling and nitric oxide production (131, 132). Increased neutrophil adhesion in diabetes has predominantly been investigated in the context of vascular sequalae and is therefore outside the scope of this review (129, 130, 133).

## Neutrophil ROS Production

In health, ROS production in neutrophils is tightly regulated, since reduced or increased production impacts infection resolution and tissue integrity respectively (134, 135). Neutrophil ROS production, *via* the nicotinamide adenine dinucleotide phosphate (NADPH) oxidase complex, is significantly impacted by hyperglycaemia in diabetes and has been extensively studied previously in animal models, healthy volunteers, and those with type 1 or T2D (**Table 2**). Formation of the NADPH oxidase complex occurs at the phagosomal membrane for intracellular killing of pathogens and at the plasma membrane for extracellular ROS release (161, 162).

**Table 2:** Studies Investigating Neutrophil ROS production in Diabetes.

Study	Animal model/human volunteer type	Changes in neutrophil ROS production reported in diabetes group compared to healthy control
**Studies investigating neutrophil extracellular ROS production**		
(54)	HVs + people with T2D	↑ in response to PMA and zymosan
(136)	HVs + people with T2D	↑ in response to PMA ↓ in response to zymosan
(137)	HVs + PWD (does not specify type)	No difference in response to PMA
(138)	HVs + people with T1D	↑ in unstimulated neutrophils. ↓ in response to fMLP and no difference when using PMA
(55)	Akita mouse (point mutation in Ins2 gene- inability to produce insulin-T1D model)	↑ in response to fMLP
(139)	HVs+ well controlled T1D	No difference in response to PMA
(140)	HVs+ volunteers with poor, moderate or well controlled T1D or T2D	↑ in response to fMLP in poorly controlled diabetes only (>8% HbA1c)
(123)	Low dose STZ-treated mice vs. WT	↓ in response to PMA
(141)	Healthy cats vs. diabetic cats (partial pancreatectomy)	↑ in response to PMA
(142)	HVs + patients with diabetes (T1D or T2D)	↑ in unstimulated neutrophils but decreased in response to PMA and zymosan
(143)	HVs + people with poorly controlled T2D	↓ in response to a mixture of zymosan, phorbol and NaF
(144)	HVs + patients with odontogenic bacterial infections or oral candidiasis with or without diabetes	↓ in response to PMA
(59)	HVs+ people with T1D or T2D with and without varying severities of periodontitis	↑ ROS in response to PMA and fMLP in participants with moderate (7-8%) or poor (>8%) glucose control
(145)	HVs + people with DFD	↓ in ROS in response fMLP. G-CSF increased ROS.
***Studies investigating neutrophil intracellular ROS production***		
(122)	HVs + people with T2D undergoing tooth extractions	↓ in ROS (stimulus not reported)
(146)	HVs + people with T2D and varying stages of diabetic nephropathy	↑ ROS. Greatest increase in patients with stage 4 nephropathy. (Multiple stimuli employed)
(147)	Newly diagnosed T1D patients not yet undergoing insulin therapy, T1D patients with disease duration of >3 months and healthy controls	↓ in ROS in response to PMA (greatest decrease in patients without insulin therapy)
(148)	HVs + people with T1D or T2D	↓ in ROS in response to PMA. Tolrestat increased ROS
(149)	HVs + infection free people with poorly controlled T2D (HbA1C <7.5%)	↓ in ROS in response to PMA
(126)	HVs + people with T2D and periodontitis	No difference in response to PMA. ↓ in response to zymosan
(150)	STZ-treated rats v.s WT rats	↑ ROS at basal level (no stimulus used)
(125)	HVs + people with diabetes and periodontal disease	No difference in response to PMA. ↓ in response to zymosan
(49)	HVs + People with T1D or T2D	No difference in response to PMA
(151)	HVs + people with T1D or T2D	↓ in response to endotoxin activated plasma
(141)	Healthy cats & diabetic cats (partial pancreatectomy)	No difference in response to PMA
***Studies investigating neutrophil intracellular and extracellular ROS production using chemiluminescence***		
(142)	HVs + patients with diabetes (T1D and T2D)	↑ in unstimulated neutrophils but decreased in response to PMA and zymosan
(152)	HVs + people with T2D	↓ in response to PMA
(153)	WT Wistar rats v.s STZ treated rats	↓ in response to fMLP
(154)	HVs + people with T1D or T2D	↓ in response to PMA
(155)	WT Fisher Rats + STZ treated rats	↑ in response to bradykinin
(143)	WT Wistar rats v.s STZ treated rats	↓ in response to opsonised zymosan
(151)	HVs + people with T1D or T2D	↑ in response to opsonised zymosan
(156)	HVs + people with T2D	↓ in response to fMLP
(157)	HVs + people with T1D and T2D	↑ ROS in response to cAMP-elevating agent- dibutyryl cAMP
(158)	Healthy wistar rats v.s alloxan treated rats	↑ ROS in response to PMA
(159)	HVs + people with T2D	↑ ROS at rest and in response to PMA
(49)	HVs + people with T1D or T2D	↓ ROS in response to opsonised zymosan and PMA
(160)	HVs+ poorly controlled T1D patients	↓ ROS in response to PMA

HVs, Healthy volunteers; fMLP, N-Formyl-methionyl-leucyl-phenylalanine; cAMP, cyclic adenosine monophosphate; NaF, Neutrophil activating factor; WT, Wild-type; G-CSF, Granulocyte colony stimulating factor.

## Extracellular ROS Production and Oxidative Stress

Although laboratory assays that detect extracellular ROS in isolation, such as the ferricytochrome c assay, are hampered by technical limitations, this technique has shown that superoxide production was significantly higher in those with diabetes and poor glycaemic control, compared to well controlled diabetes and healthy controls (**Table 2**) (59, 163). This was concomitant with a significant increase in PKC activity and DAG (59). There are previous studies showing no difference or reduced extracellular ROS production in diabetes and due to large differences in experimental design between studies it is difficult to discern the cause of conflicting evidence in the field, but may be due to differences in the ROS inducer employed and the variations in animal and human subjects used (123, 144, 145). Overall, the weight of evidence supports increased ROS production in neutrophils, as additional studies using chemiluminescent based assays that detect both intracellular and extracellular ROS, found increased levels in human subjects with T1D or T2D (151, 152, 158, 159).

Increased ROS, together with decreased activity of the ROS scavenging enzyme superoxide dismutase, is not uncommon in diabetes and the use of antioxidants to reduce excess ROS in experimental models of diabetes has been explored (158, 164, 165). The antioxidant, astaxanthin, significantly reduced extracellular ROS from neutrophils in alloxan treated rats at baseline, but not when stimulated with artificial ROS inducer Phorbol 12-myristate 13-acetate (PMA) (158). Astaxanthin also significantly lowered ROS in retinal and pancreatic cells in rats with STZ-induced diabetes and is currently undergoing clinical trials for treating diabetic retinopathy (NCT03702374) (166–168). A further antioxidant Captopril, an angiotensin-converting enzyme inhibitor, was shown to be effective in reducing ROS in human subjects with T2D and STZ-treated rats *in vitro* (155, 169).

## Intracellular ROS Production

Unlike extracellular ROS, intracellular ROS is most often reported to be significantly decreased in neutrophils in studies of T1D or T2D, which is thought to contribute to susceptibility to infection (**Table 2**) (49, 154, 170). This was demonstrated in both a model of *S. aureus* hind paw infection in leptin deficient diabetic mice, and a model of polymicrobial bacterial sepsis in obese diet-induced diabetic mice (model of T2D) (65, 109). Reduced levels of ROS were associated with lower bacterial clearance and increased mortality (65, 109).

Hyperglycaemia causes reduced ROS production due to the molecular shunting of excess glucose from glycolysis to the polyol pathway, which increases the requirement for NADPH and thereby reducing the availability to produce ROS (171, 172). Multiple approaches to increase neutrophil ROS production have been studied including *via* Tolrestat and Epalrestat, which are inhibitors of aldose reductase, a key enzyme in the polyol pathway. The inhibitors significantly increased ROS in neutrophils in both human and rodent models of diabetes (148, 171, 172). Also, enhancing ROS using granulocyte-colony stimulating factor (G-CSF) was effective in increasing ROS in multiple studies of patients with DFD (145, 173, 174). Interestingly, a Cochrane review of 5 randomised controlled trials (RCTs) with a total of 167 patients concluded that G-CSF should not be recommended as an adjuvant to current therapies for treating DFD, as it did not improve infection resolution (175). However, G-CSF treatment did reduce the need for surgical interventions and length of hospital stay in some studies. The RCTs reviewed were graded as low quality and were not statistically powered to robustly explore treatment differences. Further, large scale RCTs were recommended (175). An alternative therapeutic approach to increasing neutrophil ROS production was demonstrated in a placebo-controlled clinical study of 30 patients with T2D, using the NADPH precursor nicotinamide (149). However, until recently, there has been little, if any, further research in this area, perhaps as a result of an increased focus on reducing oxidative stress to manage diabetic complications as opposed to increasing ROS to aid pathogen handling. Nonetheless, enhancing neutrophil ROS production in sepsis is a novel clinical context where this therapeutic approach may be of value. The administration of granulocyte macrophage- colony stimulating factor (GM-CSF), significantly increased ROS production and survival in obese diabetic mice with sepsis (65). People with diabetes and sepsis have a worse prognosis than those with sepsis alone, and enhancing ROS production for acute infections, may outweigh the negative impacts of oxidative stress long term (176).

## Neutrophil Extracellular Trap Formation

As a last resort in the anti-microbial defence strategy neutrophils undergo a programmed cell death known as NETosis, in order to capture extracellular bacteria. The release of NETs, which are extruded DNA networks decorated with antimicrobial proteins and histones, can occur *via* ROS dependant or ROS independent pathways (177–180). Chromatin decondensation, a key part of NETosis, is mediated by protein arginine deiminase 4 (PAD-4), which citrullinates DNA, as well as myeloperoxidase and neutrophil elastase (181–183). Elevated NETosis damages host tissue and exacerbates inflammation and is widely investigated in the pathology of multiple chronic diseases including chronic obstructive pulmonary disease (COPD) (184, 185). In diabetes, NETs are implicated in disease complications, contributing to poor infection resolution in DFD, retinopathy and cardiovascular sequalae, as well in the early pathophysiology of T1D (186–189). NETosis is increased in the presence of high glucose concentrations and is consistently shown to be upregulated in diabetes (56, 190–193). The mechanism of hyperglycaemia induced ROS production, as discussed above, also drives enhanced NETosis in diabetes and the impacts on NETosis are durable, with elevated NET levels persisting for up to a year post normalisation of blood glucose in study of people with T2D (90, 193). Membrane bound and intracellular proteases, such as neutrophil elastase, that are externalised during the process of NETosis have greater activity in people with diabetes, further contributing to the excessive inflammation observed (194). The strong association between increased NETosis and diabetes means it is a leading area in the field for the investigation of immune-modulating therapies.

Wong et al., demonstrated for the first time that neutrophils isolated from people with T1D, T2D or STZ-induced diabetic mice were primed to undergo NETosis (56). Furthermore, wound healing was impaired in STZ treated mice, which was reversed in PAD-4 knockout mice, providing a rationale for targeting PAD-4 therapeutically to reduce NETosis (56). Fadini et al. (195) showed evidence of NETosis occurring in skin lysates of diabetic mice, and that the PAD-4 inhibitor, cl-amidine, improved wound healing in STZ-treated mice (195). However, ROS dependant NETosis does not rely exclusively on PAD-4 for NETosis, therefore different approaches to reduce ROS production have also shown positive outcomes in lowering levels of NETosis (182). Targeting PKCβ2, using ruboxistaurin improved wound healing and reduced NETosis in STZ treated mice with sterile injury (64). Anti–vascular endothelial growth factor therapy was also shown to be effective in reducing ROS-dependant NETosis in retinas from STZ-treated rats, in the context of diabetic retinopathy and has yet to be explored in the context of active infection in diabetes (Wang et al., 2019).

Pathogen, rather than host therapeutic targets also show promise. A monoclonal blocking antibody to *S. aureus* pore-forming alpha toxin (MEDI4893), significantly reduced NETosis and *S. aureus* wound burden, and increased wound resolution in TALLYHO/JngJ mice (a polygenic T2D mouse model) (196). The efficacy of MEDI4893 was supported in subsequent research and provided a novel mechanism of NETosis inhibition. Low density neutrophils (LDNs) are a sub-population of neutrophils, which have an immature nuclear structure and are associated with increased NETosis in other chronic diseases such systemic lupus erythematosus (197, 198). A significant increase in the number of LDNs and neutrophils undergoing NETosis were detected in diabetic mice (both *db/db, a T2D model*, and STZ treated mice) following systemic *S. aureus* infection compared to non-diabetic control animals (66). Interestingly, neutralising *S. aureus* alpha-toxin with MEDI4893 inhibited TGF-β-mediated induction of LDNs and NET production, and increased animal survival (66). Improving the bactericidal capacity of NETs to improve infection resolution has also been explored. Clarithromycin increased the killing capability of NETs from people with T2D by increasing the antimicrobial cathelicidin peptide, LL-37 (199). Manipulating NETosis is an expanding area of research in the field and provides a promising future avenue for immune-modulating therapies to help reduce complications of T1D and T2D and improve handling of infections.

### The importance of NETosis in COVID-19

COVID-19 caused by the novel SARS-CoV-2 virus is a complex respiratory and multi-organ syndrome characterised by respiratory distress, hyper inflammation, and coagulation. COVID-19 disproportionately impacts the elderly and those with underlying health issues, and both T1D and T2D are associated with severe COVID-19 disease and increased mortality (200–204). In those with diabetes, a high HbA1c, suggesting chronic hyperglycaemia, upon hospital admission is an independent risk factor for poor prognosis and mortality (205–208). An increased neutrophil/lymphocyte ratio predicts poor clinical outcomes in COVID-19 patients and increased NETosis is considered a key mechanism driving airway inflammation and lung damage in this disease (68, 209–211). Serum from people with COVID-19, as well as live SARS-CoV-2, induce NETosis in neutrophils isolated from healthy donors, which in turn have the capacity to cause lung epithelial cell death (69, 209). Furthermore, circulating NET markers are high in people with COVID-19 and NETs are visualised in both lung aspirates and tissue specimens (69, 212). Developing new therapies to reduce NETosis in COVID-19 is an active area of research in the midst of the global COVID-19 pandemic (69, 213). It is possible that successful anti-NETosis therapies for COVID-19 may help other chronic diseases where NETs are implicated in the pathogenesis, with diabetes being a key candidate for this.

## Neutrophil Cytokine Production

Neutrophils produce a range of pro-inflammatory and anti-inflammatory cytokines, which are integral to effective innate and adaptive immune responses (214). Neutrophils isolated from people with T2D generated significantly increased levels of pro-inflammatory cytokines; IL-8, TNF-α and IL-1β at both basal levels and when stimulated with LPS *in vitro* (215). Increased gene expression of pro-inflammatory cytokines IL-6, TNF-α and IFN-β, were demonstrated in a subsequent *in vitro* study of neutrophils isolated from people with T2D and good glucose control (HbA1c 6-7.5%) (216). However, there was not an elevated cytokine profile in the sub-group of patients with complications of diabetes such as DFD, neuropathy and nephropathy, irrespective of glucose control, with the authors suggesting a ‘burnt out’ neutrophil phenotype in those with severe complications, although a mechanism for this phenotype was not explored (216). Furthermore, there were no differences in serum cytokine levels between the T2D group and healthy controls (216). This finding is at odds with previous studies showing elevated pro-inflammatory cytokines (IL-1α, IL-4, IL-6) in serum and whole blood from both children and adults with either T1D or T2D (217, 218).

Increased pro-inflammatory cytokine generation can result from hyperglycaemia and AGEs, which drive ROS production and intracellular calcium concentration, activating NF-κB, and promoting the transcription of pro-inflammatory cytokines (219–221). Blood glucose lowering therapies; insulin, metformin and glibenclamide, were demonstrated to reduce neutrophil cytokine production in a rodent model and from neutrophils isolated from people with T2D (222–224). However, suppression of IL-1β production by neutrophils in patients receiving glibenclamide, was associated with enhanced susceptibility to *Burkholderia pseudomallei* infection in people with T2D, highlighting the need for careful consideration of unwanted side effects when seeking to modify excessive inflammation in diabetes (224). Nevertheless, the usefulness of targeting cytokine production in response to infection and inflammation is demonstrated by the efficacy of Tocilizumab, a receptor inhibitor of IL-6, used for treating rheumatoid arthritis (RA) and more recently shown to increase patient survival in severe COVID-19 (225, 226). However, limited data suggests the effectiveness of Tocilizumab was confounded in hyperglycaemic patients with COVID-19 (both with and without diabetes) and warrants further investigation to understand the potential efficacy in treating inflammation in patients with diabetes (227).

Neutrophils produce anti-inflammatory cytokines in order to downregulate inflammation. IL-1 receptor antagonist (IL-1ra) is upregulated in people with T2D compared to healthy controls, despite pro-inflammatory cytokines also being significantly increased (214, 216, 228). A RCT of 39 patients with RA and T2D were treated with anakinra, a recombinant IL-1ra (229). The primary endpoint of the study was a reduction of HbA1c, which was met with no adverse events (229). Anakinra is also under exploration for treating COVID-19 associated inflammation, with small scale trials showing clinical improvements in patients (229–231). Anakinra was demonstrated to reduce IL-1 induction of NETs *in vitro*, using cells isolated from people with pyogenic arthritis, pyoderma gangrenosum and acne (PAPA) syndrome and in human bronchial epithelial cells (232, 233). Owing to the importance of NETs in the pathology of diabetes complications, detailed exploration on the efficacy on anakinra in treating diabetes associated neutrophil dysfunction would be an important novel addition to the field.

## Neutrophil Apoptosis

Unlike NETosis, neutrophil apoptosis is an anti-inflammatory form of programmed cell death. There are two main routes to apoptosis; the extrinsic (initiated by membrane bound death receptors) and intrinsic (regulated at the mitochondrial level) pathways, which share a common mechanism of caspase mediated cell shrinkage, cytoskeleton breakdown and nuclear fragmentation (234–236). Accumulation of neutrophils at sites of inflammation, without undergoing apoptosis and clearance, causes host tissue damage and release of pro-inflammatory cytokines (237–241). Delayed neutrophil apoptosis is reported in chronic respiratory diseases such as COPD (242–244). Research in diabetes thus far presents a complex dysregulation of neutrophil apoptosis, whereby apoptosis is reduced but there is a weak response to anti-apoptotic (pro-survival) signals.

Manosudprasit et al. demonstrated reduced spontaneous apoptosis in peripheral blood neutrophils from people with T2D and those with T2D and periodontitis; a common oral infection in PWD (57). Down regulation of the key proteases involved in neutrophil apoptosis, caspases 3 and 9 were reported. Within the patient group, apoptosis was delayed significantly in those with a high HbA1C (>7.5%). However, this phenotype could not be recreated using healthy donor neutrophils incubated in high glucose media (25 mM) *in vitro.* Delayed neutrophil apoptosis was observed in non-obese diabetic mice (a T1D model) infected with *S. aureus*, which was associated with enhanced production of TNF-α (58). Elevated levels of TNF-α are implicated in the aetiology of chronic wounds in diabetes and periodontitis (245, 246). In contrast, neutrophils from people with diabetes do not have a cell survival advantage in response to lipopolysaccharide (LPS), potentially enhancing susceptibility to infection in humans (47, 247, 248). LPS is a cell wall component of Gram-negative bacteria and is a well characterised pro-survival stimulus in neutrophils (239, 249). LPS tolerance, in which cells become less responsive to LPS, is observed in Goto-Kakizaki rats (a T2D model), and mediated by impaired Toll-like-receptor 4 (TLR4) signalling (47, 250) which may have profound consequences on immune responses to infection.

Furthermore, the impact of delayed apoptosis is exacerbated in diabetes due to reduced macrophage efferocytosis (251, 252). Promoting neutrophil apoptosis as an anti-inflammatory strategy has been successfully demonstrated in experimental models of other chronic diseases including COPD and in human studies *in vitro* (150, 244, 253–256). However, this therapeutic approach has not been widely investigated in diabetes. Limited data demonstrates 1,25-dihydroxy-vitamin-D3 (1,25VitD3) increased apoptosis *in vitro* in people with T2D and periodontitis and presents an area where additional research is warranted (257).

## Neutrophil Phagocytosis

Neutrophil phagocytosis is the engulfment and internalisation of organisms into membrane bound compartments (phagosomes) prior to pathogen killing. Phagocytosis phenotypes have been widely investigated in diabetes previously, predominantly in small scale studies using volunteers with T1D or T2D and rodent models (**Table 3**). The weight of evidence demonstrates a reduction in neutrophil phagocytosis in response to a variety of stimuli (123, 147, 264, 267). However, some studies report no difference in phagocytosis by neutrophils from PWD in comparison to healthy controls (49, 145, 268). The majority of studies reviewed do not recruit treatment naïve PWD, and therefore it must be assumed that some participants will be prescribed standard anti-hyperglycaemic therapies. Insulin therapy restored blood glucose and neutrophil phagocytosis, in leptin-deficient mice, highlighting the importance of accounting for current treatments when designing studies using human volunteers (271). Hyperglycaemia elevates intracellular calcium levels as a result of cell shrinkage in response to osmotic stress, which in turn dysregulates cellular signalling mechanisms required for actin rearrangement in phagocytosis (74). Inhibiting uptake of calcium, using the calcium ion channel blocker, amlodipine (a treatment for hypertension and coronary heart disease) increased neutrophil phagocytosis in patients with T2D (263, 272). Furthermore, high glucose interferes with complement protein C3 mediated opsonisation of *S. aureus* and *Candida albicans*, which could further add to reduced neutrophil phagocytosis and pathogen handling in T1D and T2D (273, 274).

**Table 3:** Studies investigating neutrophil phagocytosis in diabetes.

Study	Animal model/human volunteer type	Phagocytosis phenotypes reported in diabetes
***Studies reporting a decrease in neutrophil phagocytosis in diabetes compared to control***		
(258)	HVs + peoplewith T2D	↓ in phagocytosis of *S. aureus*- only acidotic diabetic group, no difference in people with non-acidotic diabetes
(50)	Alloxan treated rat model	↓ in phagocytosis of *Streptococcus pneumoniae*
(259)	HVs + children with T1D	↓ in phagocytosis
(260)	HVs + T2D	↓ in phagocytosis of *S. aureus* but no difference in phagocytosis of *S. epidermidis*
(261)	HVs + people with T2D	↓ in phagocytosis of *Burkholderia pseudomallei*
(262)	Alloxan and diet induced diabetic mice	↓ in phagocytosis of LPS-coated fluorescent beads
(190)	Alloxan treated rats- peritoneal neutrophils	↓ in phagocytosis of opsonised *Candida albicans*
(65)	Abdominal sepsis model in diabetic diet induced mice	↓ in phagocytosis of *Escherichia coli*
(263)	HVs + people with T2D and poorly controlled blood glucose (>120 mg/dL)	↓ in phagocytosis of oil droplets containing oil red O, coated with *E. coli* derived LPS
(264)	HVs + people with T2D	↓ in phagocytosis of opsonised oil droplets containing oil red O, coated with *E. coli* derived LPS
(123)	Low dose STZ-treated mice	↓ in phagocytosis of zymosan
(265)	WT mice v.s db/db mice	↓ in phagocytosis of pHrodo Red *S. aureus* Bioparticles Conjugate
(150)	STZ-treated rats v.s WT rats	↓ in phagocytosis of opsonised and unopsonised *Saccharomyces cerevisiae*
(147)	Newly diagnosed T1D patients not yet on insulin therapy, T1D patients with disease duration of >3 months and healthy controls	↓ in phagocytosis of *E. coli* (greatest decrease in new diagnosed patients, not undergoing insulin therapy)
(122)	HVs + people with T2D undergoing tooth extractions	↓ in phagocytosis of FITC- labelled opsonised *E.coli*
(266)	HVs + people with diabetes controlled with insulin	↓ in phagocytosis *Candida guilliermondii*
(139)	HVs + people with well-controlled T1D	↓ in phagocytosis *Candida albicans*
(267)	HVs + people with T2D	↓ in phagocytosis- only in K1/K2 *Klebsiella pneumoniae* no difference in non-K1/K2 serotypes
(151)	HVs + people with T1D or T2D	↓ in phagocytosis of heat killed opsonised *Candida albicans*
(154)	HVs + people with T1D or T2D all receiving insulin	↓ in phagocytosis of *S. aureus*
(144)	HVs + patients with odontogenic bacterial infections or oral candidiasis with or without diabetes	↓ in phagocytosis of latex particles
***Studies reporting no difference in neutrophil phagocytosis in diabetes compared to control***		
(268)	HVs + people with T1D	No difference on phagocytosis of *Candida albicans*
(49)	HVs + people with T1D or T2D	No difference when using C3 opsonized latex beads
(145)	HVs + diabetic patients with active foot infection	No difference in phagocytosis of *S. aureus*
(109)	*S. aureus* hind paw infection model in db/db mice	No difference in phagocytosis of *S. aureus* (bone-marrow derived neutrophils)
(269)	HVs+ poorly controlled diabetes (HbA1c ≥ 10%)+ well controlled diabetes (HbA1c < 7%) + morbidly obese+ obese with metabolic syndrome + obese without metabolic syndrome	No difference in uptake of opsonised *S. aureus*
(149)	HVs + infection free people with poorly controlled T2D (HbA1C <7.5%)	No difference in phagocytosis of *S. aureus* but a downward trend reported
(70)	HVs +people with T1D + latent autoimmune diabetes in adults + people with T2D	No difference in phagocytosis of FITC-labelled zymosan
(270)	Akita mice lacking p47^phox^ (Akita/Ncf1) (model of periodontitis and chronic hyperglycaemia)	No difference in the phagocytosis of FITC- labelled zymosan *(in vivo)*
(52)	Lean zucker rat v.s obese zucker rat (T2D model) (peritoneal neutrophils)	No difference in the phagocytosis of *C. albicans*

HVs, Healthy volunteers; WT, wild-type; FITC, Fluorescein Isothiocyanate.

Once phagocytosis is complete and bacteria are contained within the phagosome, phagosomal maturation occurs in order to conclude the killing process. Here the phagosome fuses with intracellular granules to form a phaglysosome, into which antimicrobial granule contents are discharged. Furthemore, the phagolysosome becomes acidified, which is required for effective pathogen killing (275). Phagosomal maturation has not been studied widely in the context of diabetes previously. Limited data in a db/db mice model, showed a significant reduction in phagosome maturation and killing of *S. aureus* compared to control mice and maturation was augmented by insulin treatment (271). Phagosomal maturation relies on glycolysis, and perturbations of glycolysis, mediated by pathogens including *Salmonella typhimurium* have been demonstrated to reduce acidification and bacterial killing in macrophages from healthy volunteers (276, 277). Reduced activities of key glycolytic enzymes (G6PDH and glutaminase) were demonstrated in STZ treated rats, and whether aberrant glycolysis is an important factor in phagosomal maturation is yet to be investigated in T1D or T2D (278). Furthermore, complement protein C5a, was shown to impact phagosomal maturation *via* phosphoinositide 3-kinase (PI3K) signalling in neutrophils from critically ill patients in response to *S. aureus* challenge *in vitro* (279). Further research is required to explore whether phagosomal maturation is a fundamental defect in those with T1D or T2D and to elucidate the causative mechanisms.

## Impacts of Hypoglycaemia on Neutrophil Response to Infection

Iatrogenic hypoglycaemia remains one of the major challenges in the treatment of T1D and T2D (280). Data from self-reporting studies, which are likely to be underestimates, suggest people with T1D have approximately two hypoglycaemic episodes per week, with an annual incidence of severe hypoglycaemia, where third party assistance is needed, being 1.15 events per person per year in T1D versus 0.35 events per person per year in T2D (281, 282). Mechanistic studies employing the hyperinsulinaemic-hypoglycaemia clamps in both healthy individuals and those with T1D and T2D, demonstrate that acute moderate hypoglycaemia initiates a pro-longed pro-inflammatory state with upregulation of C-reactive-protein (CRP), increased platelet reactivity and mobilisation of pro-inflammatory leukocyte subsets (283–286). Additionally, in response to low endotoxin challenge in healthy volunteers, neutrophil counts were significantly increased in those allocated to experimental hypoglycaemia 48 hours earlier when compared to euglycaemic controls (284). However, whether neutrophils released into the circulation in response to hypoglycaemia have an altered function has not been widely investigated. A small-scale study compared the neutrophil oxidative burst in response to *S. aureus* in people with T1D versus healthy controls, after an insulin induced hypoglycaemic episode (287). A greater reduction in oxidative burst was shown in the healthy control group compared to those with T1D (287). Sub-populations of PWD are more prone to hypoglycaemic events; including older people with multiple co-morbidities such as chronic kidney and liver disease, those with a long disease duration, people treated with insulin and sulfonylureas, those with impaired awareness of hypoglycaemia and individuals with low c-peptide levels (288–290). Investigating neutrophil function in observational cohorts susceptible to hypoglycaemia in both T1D and T2D, could provide novel insights into the impacts of hypoglycaemia on neutrophil function in the free-living condition. Notwithstanding potential confounding factors from unmeasured variables, these data could be highly relevant in understanding the effects of hypoglycaemia on neutrophil function in a ‘real-world’ setting. This is because existing literature on neutrophil function from hyperinsulinaemic-clamp studies is limited by supraphysiological doses of intravenous insulin used that are almost never encountered in routine clinical practice and insulin at these levels is known to exert strong inflammatory effects (291, 292).

## Discussion

**Figure 2:**
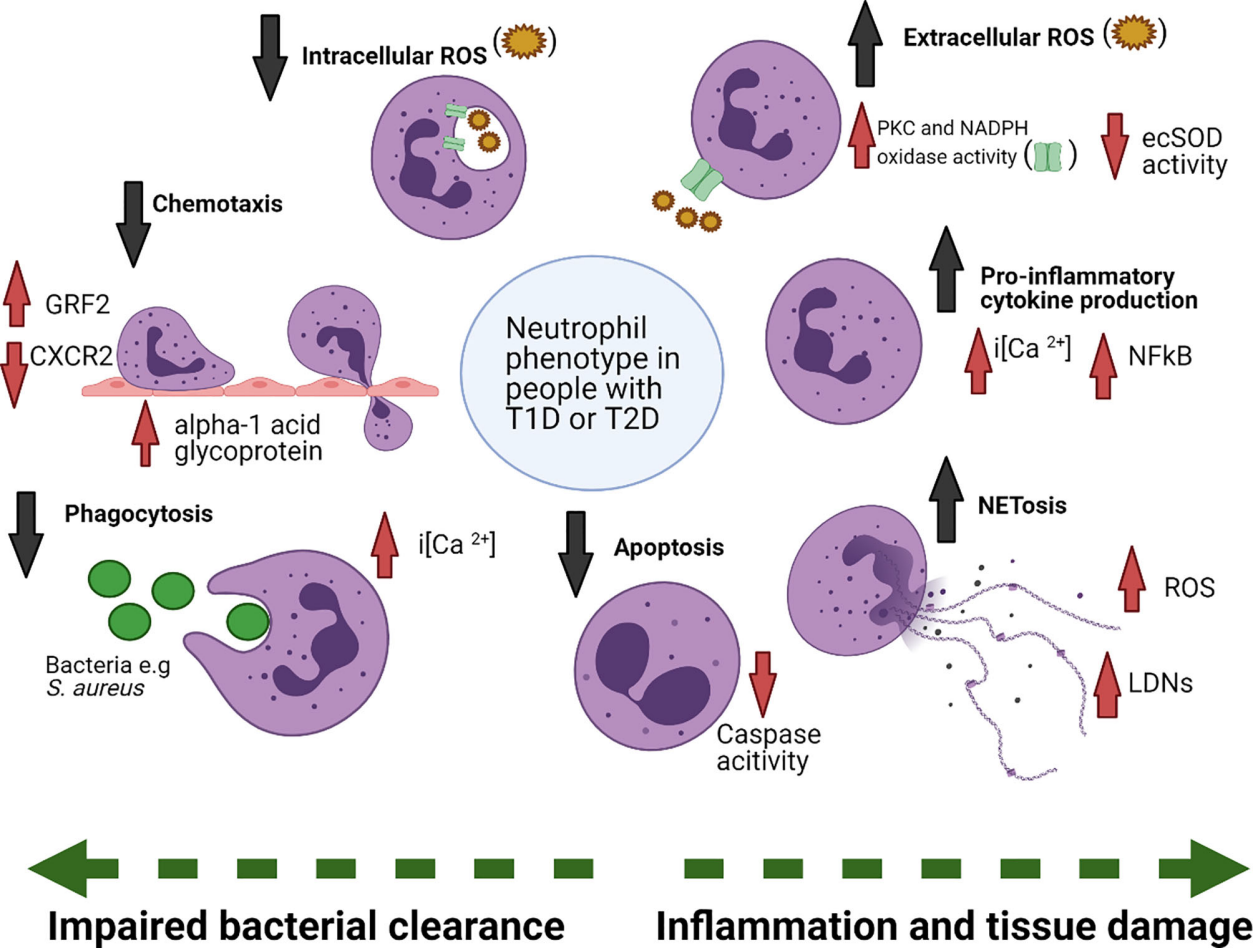
Summary of changes in neutrophil function in diabetes. Neutrophils in diabetes are functionally altered, due to exposure to the diabetic microenvironment, including changes to blood glucose as well as other factors. Phagocytosis, chemotaxis, intracellular ROS production and apoptosis are reduced in diabetes, whereas extracellular ROS, cytokines and NETosis are increased. Examples of mechanisms underpinning the functional changes are also noted. extracellular superoxide dismutase (ecSOD), protein kinase C (PKC), nicotinamide adenine dinucleotide phosphate (NADPH), reactive oxygen species (ROS), Nuclear factor-κB (NF-κB), low-density neutrophils (LDNs), G protein coupled receptor kinase-2 (GRK2). Figure created with BioRender.com.

Increased susceptibility to recurrent and chronic infections is a key clinical characteristic of both T1D and T2D. This literature review has highlighted that there is an abundance of small-scale studies observing phenotypic changes between either human volunteers or animal models with and without diabetes, conducted over the last 60 years (summarised in **Figure 2**). Analysis of the breadth of literature demonstrates that neutrophils in those with T1D or T2D are fundamentally altered compared to neutrophils from healthy donors. Neutrophil effector mechanisms pertinent to infection and inflammation are aberrant in diabetes. Key neutrophil pathways critical in the response to infection (recruitment, chemotaxis, phagocytosis and intracellular ROS production) are impaired in diabetes, whereas pro-inflammatory cytokine production, extracellular ROS production, cell survival and NETosis, are upregulated, and are emerging as critical mediators of diabetic complications (47, 56, 122, 140, 147, 192). An avenue of under explored research is the investigation of tissue resident neutrophils. This is particularly important in DFD which is characterised by vascular and infectious tissue pathologies. Skin lesions from *in vivo* diabetes models have provided evidence for the presence of NETs in the tissue, which supports a role for the diabetic tissue environment in modifying neutrophil function (56, 195). Tissue neutrophils in other diseases have demonstrated to have tissue specific phenotypes (293, 294). For example, increased release of neutrophil elastase was shown in neutrophils isolated from bronchial lavage fluid but not in circulating neutrophils from children with cystic fibrosis (295). Phenotyping tissue neutrophils is challenging, particularly in light of their sensitivity to *ex vivo* manipulation and short lifespan, but doing so would allow us a greater understanding of the specific role of the tissue microenvironment in modifying neutrophil function in diabetes.

Hyperglycaemia and AGEs are the key drivers of altered neutrophil function in diabetes, with dysregulated neutrophil function found in patients despite anti-hyperglycaemic therapies (90, 192). However, there are gaps in the mechanistic understanding yet to be explored. Epigenetic modification of neutrophils in T1D or T2D has not been addressed previously and could provide further insight as to how hyperglycaemia may impact neutrophil function beyond what is already understood, potentially identifying new therapeutic targets to treat dysfunction. Also, whether hypoglycaemia in diabetes can alter neutrophil function is not known. Furthermore, a pro-inflammatory neutrophil phenotype is not unique to diabetes and aberrant neutrophil function is implicated in multiple contexts including COPD and critical illness (296–298). PWD often have comorbidities which may also contribute to neutrophil dysfunction and understanding common drivers of neutrophil function maybe a useful approach for future research.

To build upon the wealth of phenotypic data already collected, future research should focus on conducting RCTs. Limited previous research shows that therapeutic modulation of dysregulated neutrophil functions can restore host immunity and improve infection resolution in diabetes (64, 65, 196). The therapeutic reduction of pro-inflammatory ROS production and NETosis is the main direction of emerging research, with positive effects on infection resolution demonstrated in small scale animal and patient research (64, 66, 158). Furthermore, the exploration of investigative therapies shown to be useful in modulating neutrophil function in other diseases such as sepsis and RA should be prioritised in diabetes. For example, anakinra, the IL-1ra antagonist targeting neutrophils in RA could be useful in reducing chronic inflammation in diabetes (232, 233). The urgency and necessity of continued research and development in the field is exemplified by the susceptibility of PWD to develop life-threatening acute respiratory distress syndrome (ARDS) with COVID-19 (299). Increased ROS and NETosis are drivers of alveolar oedema, which is characteristic of ARDS and therapies reducing these mechanisms would be of huge value (300). To conclude, future research should focus on driving forward investigation of novel experimental treatments targeting neutrophil induced oxidative stress and increased NETosis in diabetes, with the aim of conducting RCTs to translate the abundance of previous phenotypic research into effective treatments to improve the lives of people with T1D and T2D.

## Search Strategy

The following search strategy was used for this review. Literature searches were conducted using the PubMed database (1964–2020). Key word searches included ‘Diabetes’ and ‘Neutrophil’ and then either ‘Recruitment’, ‘Cytokines’ ‘Chemotaxis’,‘Phagocytosis’, ‘Reactive oxygen species’, ‘ROS’, ‘NETosis’, ‘Apoptosis’, ‘hypoglycaemia’, ‘hyperglycaemia. or ‘epigenetics’. All articles that were found using the search terms were included.

## Author Contributions

RD performed extensive literature searches, created tables and figures and wrote the first draft of the review. AI, SH, IS, and LP wrote individual sections as well as reviewed and edited the review drafts. All authors contributed to the article and approved the submitted version.

## Funding

PhD Studentship (The University of Sheffield) and Medical Research Council AMR cross-council funding to the SHIELD consortium “Optimising Innate Host Defence to Combat Antimicrobial Resistance” (MRNO2995X/1). AI is supported by a National Institute for Health Research (NIHR) Academic Clinical Lectureship.

## Conflict of Interest

SH undertakes consultancy for Eli Lilly, Sanofi Aventis, NovoNordisk, Zealand Pharma, and have been on speaker panels for NovoNordisk and Astra Zeneca. These companies’ products have effects on hypoglycaemia when treating individuals with diabetes and are therefore related to this paper. AI has consulted for OrbiMed LLC and received educational grant support from Sanofi S.A.

The remaining authors declare that the research was conducted in the absence of any commercial or financial relationships that could be construed as a potential conflict of interest.
